# Older Koreans' Information Support and Internet Use: Internet Skills and Technology Attitudes

**DOI:** 10.1093/geroni/igab046.2487

**Published:** 2021-12-17

**Authors:** Hye Soo Lee

**Affiliations:** Oregon State University, Corvallis, Oregon, United States

## Abstract

While older Koreans have growing access to Internet, they still lag in actual utilization. This study examined effects of different information support sources on Internet utilization and whether these were mediated by Internet skills and technology attitudes among older men and women. This study used secondary data from 2019 Digital Divide Survey conducted by National Information Society Agency of Korea. The sample consisted of 1,031 Korean Internet users aged 60+, including 495 men and 536 women. Support sources included personal and professional. Skills were measured by ability to use specific features of mobile devices such as smartphones (seven items), while utilization was measured by the use of mobile devices for specific reasons (25 items). Serial mediation analyses using both skills and attitudes were conducted separately according to gender and support sources, covarying for demographics and health. In general, information support was positively associated with utilization. For men, personal informational support was mediated by technology attitudes only. For women, professional informational support was mediated by both Internet skills and technology attitudes, but the serial indirect effect was not significant for this model. The other two models showed significant serial mediation effects through Internet skills and technology attitudes, in this order. Only women had significant direct associations between information support and Internet utilization. Regardless of the source, informational support is positively associated with older Koreans’ Internet utilization. Professional support for men and personal support for women may be most beneficial for greater Internet utilization.

